# Safety attitudes culture remain stable in a transplant center: evidence from the coronavirus pandemic

**DOI:** 10.3389/frtra.2023.1208916

**Published:** 2023-09-26

**Authors:** Chi Zhang, Sena Wilson-Sheehan, Brianna Ruch, Josiah Wagler, Ali Abidali, Elisabeth S. Lim, Yu-Hui Chang, Christopher Fowler, David D. Douglas, Amit K. Mathur

**Affiliations:** ^1^ Transplant Center, Mayo Clinic, Phoenix, AZ, United States; ^2^ Robert D. and Patricia E. Kern Center for the Science of Health Care Delivery, Mayo Clinic, Rochester, MN, United States; ^3^ Quantitative Health Sciences, Mayo Clinic, Phoenix, AZ, United States

**Keywords:** safety culture, transplant outcomes, postoperative outcomes, COVID, solid organ transplantation

## Abstract

**Background:**

We sought to understand how safety culture may evolve during disruption, by using the COVID-19 pandemic as an example, to identify vulnerabilities in the system that could impact patient outcomes.

**Methods:**

A cross-sectional analysis of transplant personnel at a high-volume transplant center was conducted using the Safety Attitudes Questionnaire (SAQ). Survey responses were scaled and evaluated pre- and post-COVID-19 (2019 and 2021).

**Results:**

Two-hundred and thirty-eight responses were collected (134 pre-pandemic and 104 post-pandemic). Represented organ groups included: kidney (*N* = 89;38%), heart (*N* = 18;8%), liver (*N* = 54;23%), multiple (*N* = 66;28%), and other (*N* = 10;4%). Responders primarily included nurses (*N* = 75;34%), administration (*N* = 50;23%), and physicians (*N* = 24;11%). Workers had high safety, job satisfaction, stress recognition, and working conditions satisfaction (score >75) both before and after the pandemic with overlapping responses across both timepoints. Stress recognition, safety, and working conditions improved post-COVID-19, but teamwork, job satisfaction, and perceptions of management were somewhat negatively impacted (all *p* > 0.05).

**Conclusions:**

Despite the serious health care disruptions induced by the pandemic, high domain ratings were notable and largely maintained in a high-volume transplant center. The SAQ is a valuable tool for healthcare units and can be used in longitudinal assessments of transplant culture of safety as a component of quality assurance and performance improvement initiatives.

## Introduction

A landmark report in 2,000 estimated that nearly 100,000 deaths in the United States annually were related to medical errors, calling for actions to reduce medical errors and to develop a culture of safety (1). In the wake of its publication, hospital systems worldwide sought for solutions (2–4). Many drew parallels between healthcare and the aviation industry and compared the operating room to the cockpit (5, 6). Adoption of checklists into medicine helped standardize workflows, and there was less acceptance of deviation from the norm. The field of nursing has long embraced these changes and championed for a culture of “speaking up” in the interest of patient safety (7). Overtime, fading authority hierarchies has improved healthcare safety, with evidence that high levels of hospital safety were associated with reductions in readmission rates, mortality rates, and length of stay (8–10). Consequently, improvements in patient outcomes lag the establishment of a robust safety climate.

The culture of safety requires the combined effort of all stakeholders, including nurses, physicians, patients, policy makers, and more. In the field of organ transplantation, multidisciplinary care is the norm. Collaboration between medical subspecialists, transplant surgeons, perioperative care providers, nurses, social workers, dieticians, administrative teams, and many others is required. Despite the complexity of interactions, there is a paucity of data as it pertains to transplant health worker perception of the culture of safety. Additionally, safety culture is subject to erosion by disruptive forces. External events, such as pandemics, or internal events, such as major organizational structural changes, may impact how teams function on the ground day-to-day. Their attitudes toward safe care delivery may change as a result.

We evaluated safety culture in a high volume transplant center using the Safety Attitudes Questionnaire (SAQ), a validated tool used in several health care contexts, before and during the coronavirus disease-19 (COVID) pandemic (10–12). We aim to describe the changes in worker perception of institutional safety attitudes at a single high-volume transplant center in the context of the COVID pandemic which was highly disruptive to healthcare overall. We hypothesized that the SAQ can be an effective longitudinal tool in the field of organ transplantation and used to target further quality improvement opportunities and to identify vulnerabilities within large teams.

## Materials and methods

### Data collection

A prospective survey study was conducted at a single high-volume transplant center to assess institutional culture of safety during a time of major healthcare disruption. The first survey was administered before the declaration of the public health emergency in March 2020. The second survey was administered 18 months later in 2021. All transplant staff affiliated with our high-volume multi-organ transplant center (heart, liver, kidney, pancreas transplants), including medical and surgical attending level staff, medical and surgical fellows, resident physicians, outpatient nurse coordinators from all phases of transplant, advanced transplant providers (physician assistant and nurse practitioners), pharmacists, dieticians, social workers, administrative staff, and other affiliated personnel were eligible and invited to anonymously and voluntarily complete the survey. Surveys were distributed over email to a REDCAP link. Participants were provided with two reminders for survey completion, spaced one week apart. Employees were considered non-responders if no contact was established within 30-days of initial contact. All surveys responses were digital. Employment records show that the organization supported 180 employees at the time of the first survey and 253 at the time of the second survey.

### Survey tool and data analysis

The SAQ is a 60-item questionnaire that takes approximately 10–15 min to complete on average (10). It was developed in 2006 and has been validated in many languages. It aims to assess six core factors: teamwork climate, job satisfaction, perceptions of teamwork climate, safety climate, perceptions of management, job satisfaction, working conditions stress recognition. Our version included transplant-specific questions, which were analyzed with teamwork climate questions. Each of the questions are answered in a 5-point Likert scale (1-strongly disagree to agree 5-strongly agree) with some that were negatively worded. Negatively worded questions were reverse scored. All results were linearly transformed to a score from 0 (worst) to 100 (best). Domain scores were compared before and during the COVID pandemic. Additionally, responders who scored ≥75 were compared against scores <75 both pre and intra-pandemic. This cutoff was made based on previous literature demonstrating that scores ≥75 were associated with excellent safety (10, 13, 14). Descriptive analysis was performed using ANOVA and Chi-square in demographic data. *P* < 0.05 was considered statistically significant. The study was approved by the institutional review board. Supplementary S1 contains the survey tool used for our study.

## Results

### Demographics

A total of 238 survey responses were recorded, with 134 before and 104 during pandemic. The response rates were 74% and 41%, respectively. Responders were mostly female (*N* = 183; 80%). More than half of the cohort has been working at their current position for over 3 years (*N* = 191; 63%). Nurses made up the largest group (*N* = 75; 34%), followed by administrative support staff (*N* = 50; 23%) and attending physicians (*N* = 24; 11%). The remaining groups each represented <10% of the entire cohort. Most worked within kidney transplantation (*N* = 46; 34%), followed by multiple organ transplantation (*N* = 39; 29%), liver (*N* = 32; 24%), and heart (*N* = 10; 8%). There were no distribution differences between the two compared groups (Table 1).

**Table 1:** Demographics.

	Pre COVID (*N* = 134)	Intra COVID (*N* = 104)	*P*-value
Sex, *n* (%)			0.45
Female	103 (78.6%)	81 (82.7%)	
Male	28 (21.4%)	17 (17.3%)	
Time in your current job, *n* (%)			0.90
Less than 6 months	16 (12.1%)	8 (7.8%)	
6–11 months	11 (8.3%)	9 (8.7%)	
1–2.99 years	37 (28.0%)	30 (29.1%)	
3–4.99 years	25 (18.9%)	18 (17.5%)	
5–10.99 years	26 (19.7%)	24 (23.3%)	
11–20.99 years	12 (9.1%)	8 (7.8%)	
21 or more years	5 (3.8%)	6 (5.8%)	
Primary role within Transplant center, *n* (%)			0.77
Admin support or other	27 (23.5%)	23 (22.3%)	
Advanced transplant provider	4 (3.5%)	9 (8.7%)	
Attending/staff physician	13 (11.3%)	11 (10.7%)	
Clinical social worker	9 (7.8%)	9 (8.7%)	
Clinical support or technologist/technician	9 (7.8%)	6 (5.8%)	
Dietician/nutritionist	4 (3.5%)	3 (2.9%)	
Fellow physician or resident physician	3 (2.6%)	0 (0.0%)	
Nurse manager/charge nurse or registered nurse	39 (33.9%)	36 (35.0%)	
Other management	5 (4.3%)	4 (3.9%)	
Pharmacist	2 (1.7%)	2 (1.9%)	
Organ group			0.75
Heart	10 (7.5%)	8 (7.8%)	
Kidney	46 (34.3%)	43 (41.7%)	
Liver	32 (23.9%)	22 (21.4%)	
Multiple	39 (29.1%)	27 (26.2%)	
Other	7 (5.2%)	3 (2.9%)	

**Figure 1:**
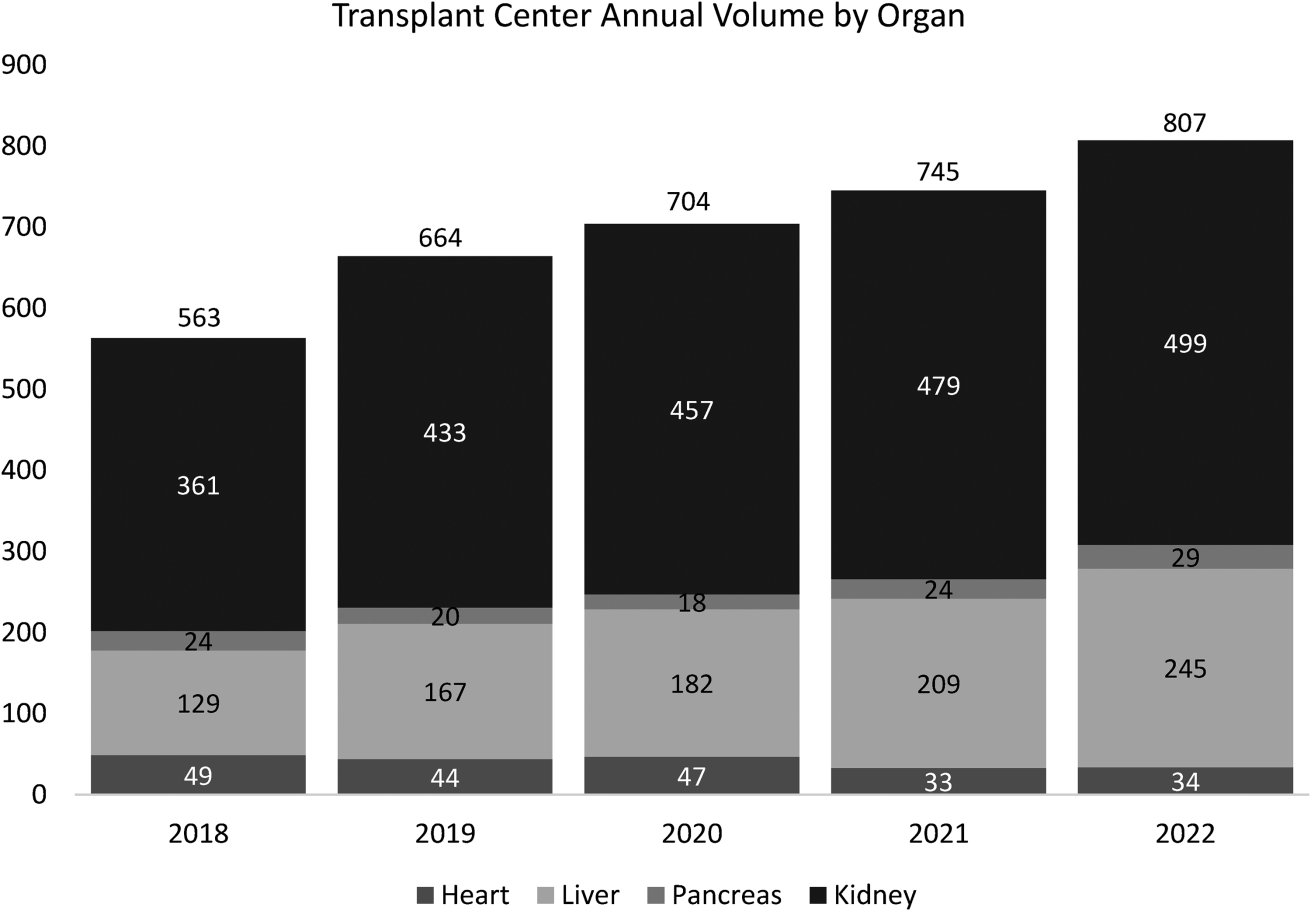
There is a steady increase in the number of organs transplanted at a single transplant center from 2018 to 2022. There was a 1.4-fold increase in the total transplant volume in 2022 (*n* = 807) compared to 2018 (*n* = 563).

Transplant center volumes increased over time from 2019 through 2021 (number of organs transplanted: 2019, *n* = 664; 2020, *n* = 704; 2021, *n* = 745). Figure 1 demonstrates the institutional transplant volume by year and by organ.

### SAQ results

Pre-COVID survey results demonstrated similarly high satisfaction across all climates. The average score was highest for safety climate (mean = 85.7; SD = 14.94), followed by job satisfaction (mean = 84.7; SD = 18.24), teamwork (mean = 83.8; SD = 15.68), perceptions of management (mean =80; SD 19.96), stress recognition (mean = 69; SD 24.25), and working conditions (mean = 64.4; SD 21.17).

**Figure 2:**
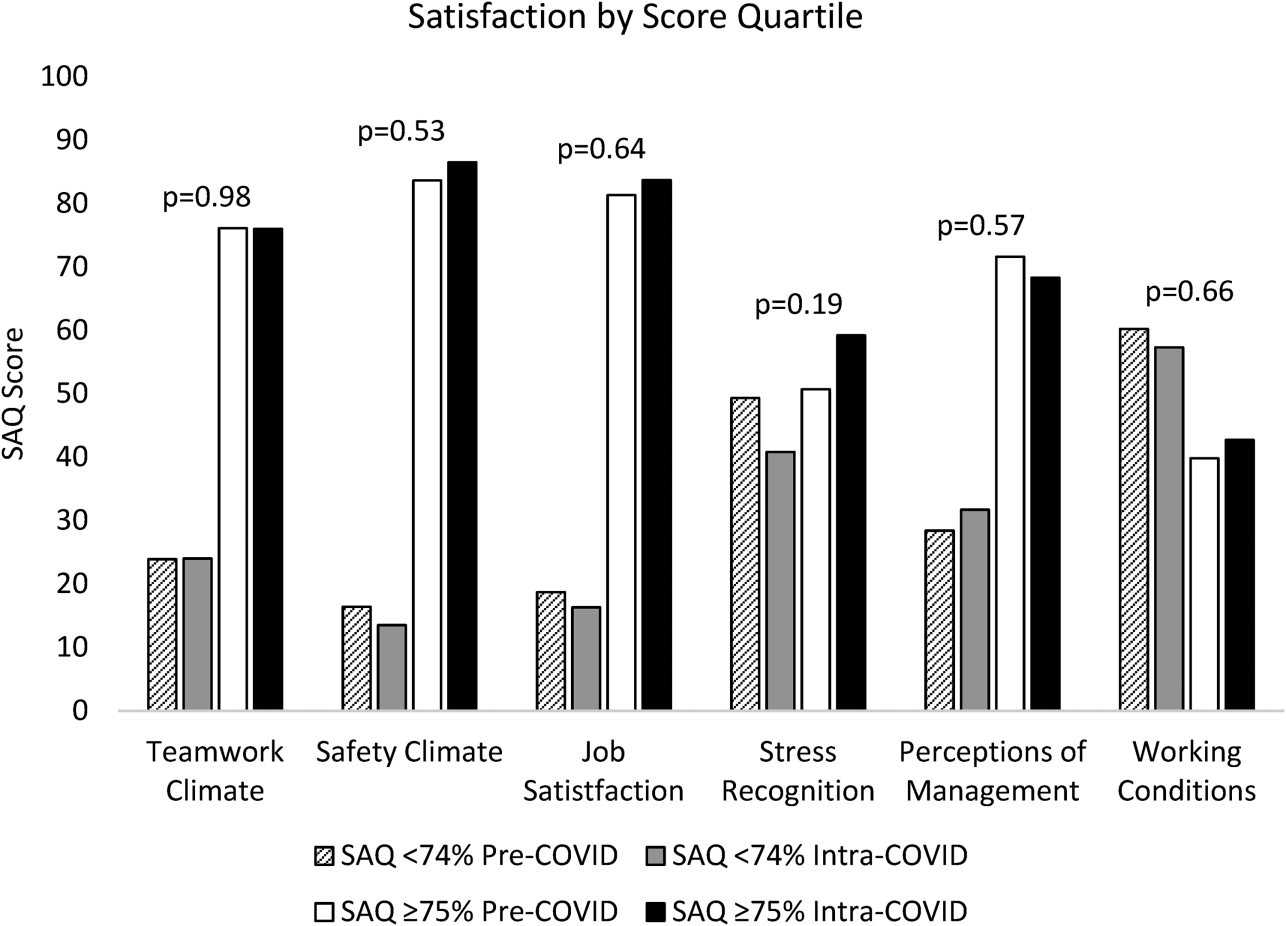
Distribution of domain-specific survey responses pre- and intra-COVID-19. Comparing the pre and intra pandemic SAQ domain scores of those top quartile scorers against the rest, there was high levels of satisfaction across five of the six domains pre-pandemic, which was stable when measured intra-pandemic.

Intra-COVID, the results across each safety climate were not significantly different, and without any ranking changes. Safety climate still had the highest satisfaction (mean = 86.1; SD = 15.12; *p* = 0.69), followed by job satisfaction (mean = 83.6; SD 19.22, *p* = 0.42), teamwork (mean = 82.9, SD = 16.43, p = 0.70), perceptions of management (mean = 77.6, SD = 22.02, *p* = 0.40), stress recognition (mean = 73.8, SD = 22.02, *p* = 0.40), and working conditions (mean = 64.9, SD 21.64, *p* = 0.75). There was lower satisfaction with perceptions of management but stress recognition and satisfaction with working conditions improved. The differences were not significant. Figure 2 shows the distribution of domain-specific survey responses pre- and intra-COVID.

Scores ≥75 were compared against those <74 both before and during the pandemic. There were more responses with a score of ≥75 within each time point and across all domains except for satisfaction with work conditions. For working conditions, 53 responses (40%) were ≥75 pre-COVID, which increased to 44 (43%) intra-COVID (*p* = 0.66). Similar increases were seen in safety climate, job satisfaction, and stress recognition (Figure 2).

**Figure 3:**
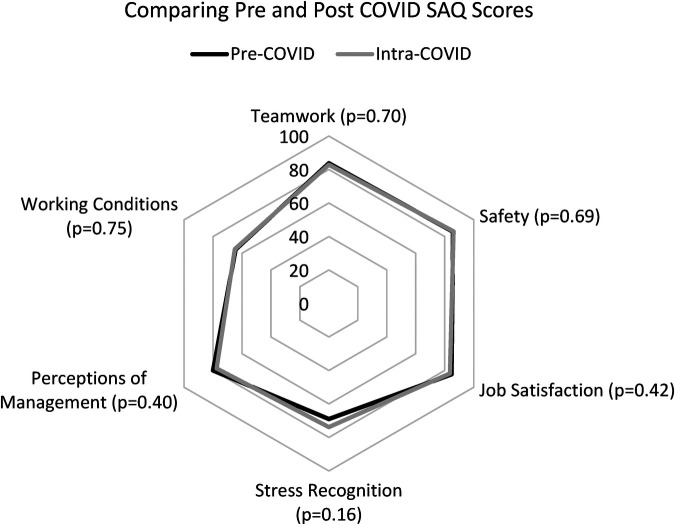
There was considerable overlap between pre- and intra-pandemic SAQ responses. The starkest difference was in stress recognition, which did not reach statistical significance.

Pre-COVID, 112 of responders (84%) scored in the top quartile for safety climate, which increased to 87% (*n* = 90; *p* = 0.53). A higher percentage of responders were also more satisfied with their jobs intra-pandemic with 84% (*n* = 87) scoring ≥75 compared to before the pandemic (*n* = 109, 81%; *p* = 0.64). Stress recognition also improved; 59% (*n* = 61) scored ≥75 for stress recognition compared to 51 (*n* = 68; 0.19). Teamwork climate satisfaction remained the same through the pandemic with 76% scoring ≥75 both pre (*n* = 102) and intra-pandemic (*n* = 79). For perception of management, the top scorers decreased from 72% (*n* = 96) to 68% (*n* = 71; *p* = 0.57). These changes were not statistically significant. Figure 3 demonstrates the degree of overlap between before and during COVID SAQ scores for individual domains.

## Discussion

Over the past few years, COVID has proved to be highly disruptive to organ transplantation, which is a gross understatement of the day-to-day reality in transplant centers. Clinical processes for deceased donors, living donors, transplant candidates, and transplant recipients had to shift monumentally overnight. Concerns about clinical outcomes and patient safety created collective anxiety experienced at all levels of transplant teams. This disruption is unprecedented, but serves as a poignant example how change can tear the fabric of transplant patient safety (15). This cross-sectional study, which measured safety attitudes in a high volume academic transplant center, demonstrated that safety culture can remain stable within transplant team during periods of disruption in health care delivery.

The SAQ has been used in multiple medical contexts including use after medical team training, the intensive care unit, trauma, pharmacy, primary care, the operating room, and in the context of the pandemic (16–20). In the era of COVID, Denning et al. showed that nurses had lower SAQ scores after the pandemic, especially in working conditions and job satisfaction categories (12). Interestingly, in contrast to our results which showed the stability of staff satisfaction, the high pre-pandemic nursing satisfaction scores were not protective of their intra-pandemic evaluation results, which the authors conclude to be largely related to increased rates of burnout and decreased supportive initiatives available. In Taiwan, a group showed substantial improvement in all metrics of the SAQ when compared to the perceptions at the beginning of the pandemic. While their results could be reflective of the recovery from early pandemic-induced pressures, they could also potentially be explained by government reduction in workload for healthcare professionals as Taiwan (and the world) transitioned out of a state of emergency (21). Clearly, global policies can dictate institutional culture of safety, and different guidelines can elicit opposite effects. Despite major changes to transplantation workflow including the limitation of transplant surgeries to life-threatening situations only, suspension of living donations, restricting procurements to local hospitals to prevent the transmission of the virus via long distance air travel, and numerous updates as we learned more about the virus, our results show that the culture of safety at our high-volume transplant center was resistant to the unprecedented pressures induced by the pandemic (22).

Our demonstrated stability across all six main domains assessed by the SAQ before and during the disruption caused by the COVID pandemic is surprising but likely attributable to several factors. First, our transplant center has evolved over time into a vast clinical enterprise with stability of personnel in leadership positions in all disciplines including medical and surgical directors, nursing, social work, and administration. Additionally, the transplant team collectively is deeply familiar with the challenges of rapid growth and high clinical volume. In fact, there was growth in transplant volume during the pandemic; in total, 664 organs were transplanted in 2019 to 704 organ transplants in 2021, and 807 in 2022. Additionally, in keeping with social distancing recommendations, the normalization of teleconferencing relieved time constraints and promoted multidisciplinary communication, allowing for consistent presence of multiple department representatives at daily morning rounds, clinical conferences such as transplant selection conferences, donor selection conferences, quality meetings, organ reviews, and departmental case reviews of adverse events. The daily gathering also facilitated the early creation of a COVID toolkit for our center as well as the ability to deliver frequent updates.

The COVID pandemic is a dramatic example of a disruption that can impact transplant care delivery. This study is highly relevant as clinical transplant teams in the United States are constantly subject to multiple types internal and external disruptions. Additionally, the fields of organ donation and transplantation are subject to the most regulation of any field in medicine. New metrics and regulation can significantly disrupt the norms of transplant care within a transplant program as it seeks to adapt to change (23). Poor performance in waitlist mortality, organ acceptance, and intra-transplant outcomes may challenge perceptions of safety within transplant programs (24, 25). New technologies and innovation may bring about several new challenges that impact safety culture (26). Also, both internal and external leadership transitions can be highly disruptive. Many other disruptions can impact transplant care delivery, but it is necessary for patient safety to maintain and improve safety culture in the face of adversity. This study demonstrates that the SAQ can be robustly applied by transplant leaders within their programs as a longitudinal model of safety cultural assessment. Culture is one of the hardest areas to change within transplant programs, and the SAQ can provide data to inform leadership and frontline staff on areas of vulnerability.

Our study is limited by its inherent survey-based nature related to reliance on self-reporting and the limited response rate, which introduces selection bias. The decrease in response rate seen intra-pandemic could be explained by the expansion of transplant employees who perhaps did not know that they were also eligible to complete the survey and could also be related to survey fatigue within the institution, work demands, and stress that precluded participation. Though it measures six distinct domains, the survey potentially suffers from the cluster effect. For example, while high scores in response to “I like my job.” indicate high job satisfaction, it may lead to positive attitudes to multiple statements from other domains such as within teamwork (“I have the support I need from other personnel to care for patients.”), safety climate (“I am encouraged by my colleagues to report any patient safety concerns I may have.”), perceptions of management (“Management supports by daily efforts.”), and working conditions (“The levels of staffing in this clinical area are sufficient to handle the number of patients.”). Despite these shortcomings, it has been validated and adapted to many other medical fields, global cultures, and different languages, though our study is the first to use it to study transplant program culture. Our findings could have also been skewed by the ceiling effect as a large percentage of responses scored ≥75 both before and during the pandemic, making differences in satisfaction between these time points difficult to detect. Despite a higher percentage of employees reporting higher satisfaction in most of the domains during the pandemic, these changes were not statistically significant. Given the potential overlap of different domains, the necessary sample size to have an adequately powered study could be considered astronomical.

Healthcare faced major disruptions and collapse of essential health services due to high COVID burden and global lockdowns. While our study takes advantage of dramatic and unexpected changes induced by the pandemic, our results can be extrapolated to other program transitions that occur as well and may be less dramatic. Additionally, though our study showed no significant changes in staff perception of the culture of safety at our high-volume transplant center despite these intrusions due to high pre-pandemic satisfaction, there is likely variation across all transplant centers in the United States. Beyond the present study, serial measurements at our own institution and at all other transplant centers has the potential to reveal areas that can be targeted for quality improvement based on perceptions of frontline staff. The implementation of a program to include serial safety culture assessments using transplant program specific SAQs could help transplant and hospital leaders develop actionable intelligence to improve care, address workforce concerns, hone processes, and ensure programs can meet the standard of efficient, highly reliable, and safe transplant care. Since transplant programs are required by regulation to be robustly engaged in quality assurance and performance improvement activities, this approach could serve as a valuable foundation for multiple initiatives. Additionally, future multi-institutional work should assess safety culture and its association with postoperative outcomes and promote generalizability of these results.

While COVID has placed unprecedented pressure on the healthcare system worldwide, the results of the SAQ revealed stability of the culture of safety at our high-volume transplant center despite external pressure. Serial examinations of transplant centers using this methodology can detect areas of vulnerability that can be actionable changes for the betterment of transplant care delivery.

## Data Availability

The raw data supporting the conclusions of this article will be made available by the authors, without undue reservation.
