# Supplementary material for: Safety attitudes culture remain stable in a transplant center: evidence from the coronavirus pandemic
Source: Front Transplant. 2023 Sep 26;2:1208916. doi: 10.3389/frtra.2023.1208916 (PMC11235290; doi:10.3389/frtra.2023.1208916)
Supplement: Supplementary file 1 [file Datasheet1.docx]

Transplant Safety Attitudes Questionnaire

Invitation to a research survey:

You are invited to participate in a research study that aims to understand the safety culture in transplant. In this study, you will be asked to complete an electronic survey (Transplant Safety Attitudes Questionnaire). Your participation in this study is voluntary and you are free to withdraw your participation at any time. The survey should only take 2 minutes to complete. All responses will be recorded anonymously. By completing and submitting this survey, you are indicating your consent to participate in the study. Your participation is appreciated.

Amit K. Mathur MD, MS

1. Nurse input is well received in this clinical area. Disagree Strongly Disagree Slightly Neutral

Agree Slightly Agree Strongly Not Applicable


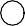

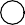

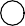

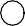

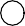

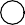


1. In this clinical area, it is difficult to speak up if Disagree Strongly I perceive a problem with patient care. Disagree Slightly

Neutral


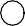

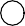

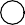

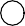

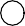

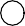


Agree Slightly Agree Strongly Not Applicable

1. Disagreements in this clinical area are resolved Disagree Strongly

appropriately (i.e., not who is right, but what is Disagree Slightly


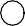

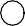

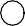

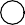

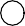

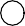


best for the patient). Neutral

Agree Slightly Agree Strongly Not Applicable

1. I have the support I need from other personnel to care Disagree Strongly for patients. Disagree Slightly

Neutral


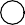

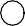

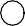

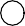

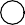

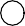


Agree Slightly Agree Strongly Not Applicable

1. It is easy for personnel here to ask questions when Disagree Strongly there is something that they do not understand. Disagree Slightly

Neutral


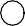

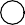

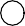

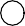

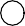

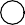


Agree Slightly Agree Strongly Not Applicable

1. The physicians and nurses here work together as a Disagree Strongly well-coordinated team. Disagree Slightly

Neutral


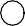

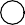

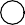

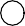

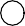

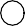


Agree Slightly Agree Strongly Not Applicable

1. I would feel safe being treated here as a patient. Disagree Strongly Disagree Slightly Neutral

Agree Slightly Agree Strongly Not Applicable


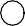

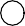

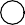

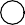

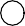

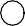


1. Medical errors are handled appropriately in this Disagree Strongly clinical area. Disagree Slightly


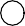

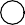

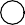

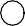

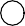

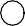


Neutral

Agree Slightly Agree Strongly Not Applicable

1. I know the proper channels to direct questions Disagree Strongly regarding patient safety in this clinical area. Disagree Slightly

Neutral


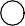

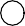

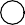

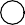

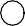

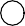


Agree Slightly Agree Strongly Not Applicable

1. I receive appropriate feedback about my performance. Disagree Strongly

Disagree Slightly Neutral


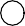

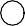

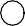

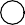

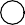

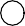


Agree Slightly Agree Strongly Not Applicable

1. In this clinical area, it is difficult to discuss Disagree Strongly errors. Disagree Slightly

Neutral


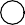

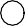

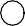

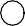

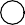

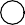


Agree Slightly Agree Strongly Not Applicable

1. I am encouraged by my colleagues to report any patient Disagree Strongly safety concerns I may have. Disagree Slightly

Neutral


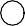

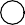

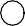

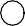

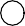

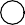


Agree Slightly Agree Strongly Not Applicable

1. The culture in this clinical area makes it easy to Disagree Strongly learn from the errors of others. Disagree Slightly

Neutral


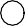

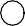

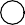

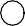

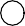

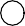


Agree Slightly Agree Strongly Not Applicable

1. My suggestions about safety would be acted upon if I Disagree Strongly expressed them to management. Disagree Slightly

Neutral


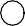

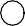

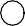

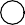

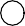

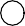


Agree Slightly Agree Strongly Not Applicable

1. I like my job. Disagree Strongly

Disagree Slightly Neutral


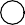

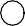

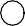

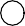

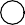

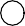


Agree Slightly Agree Strongly Not Applicable

1. Working here is like being part of a large family. Disagree Strongly Disagree Slightly Neutral


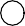

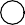

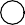

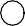

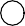

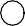


Agree Slightly Agree Strongly Not Applicable

1. This is a good place to work. Disagree Strongly Disagree Slightly Neutral

Agree Slightly Agree Strongly Not Applicable


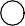

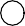

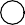

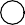


1. I am proud to work in this clinical area. Disagree Strongly Disagree Slightly Neutral

Agree Slightly Agree Strongly Not Applicable

1. Morale in this clinical area is high. Disagree Strongly Disagree Slightly Neutral

Agree Slightly Agree Strongly Not Applicable

1. When my workload becomes excessive, my performance is Disagree Strongly impaired. Disagree Slightly

Neutral

Agree Slightly Agree Strongly Not Applicable

1. I am less effective at work when fatigued. Disagree Strongly Disagree Slightly Neutral

Agree Slightly Agree Strongly Not Applicable

1. I am more likely to make errors in tense or hostile Disagree Strongly situations. Disagree Slightly

Neutral

Agree Slightly Agree Strongly Not Applicable

1. Fatigue impairs my performance during emergency Disagree Strongly situations (e.g. emergency resuscitation, seizure). Disagree Slightly

Neutral

Agree Slightly Agree Strongly Not Applicable

1. Management supports my daily efforts. Disagree Strongly Disagree Slightly Neutral

Agree Slightly Agree Strongly Not Applicable

1. Management doesn't knowingly compromise patient Disagree Strongly safety. Disagree Slightly

Neutral

Agree Slightly Agree Strongly Not Applicable

1. Management is doing a good job. Disagree Strongly Disagree Slightly Neutral

Agree Slightly Agree Strongly Not Applicable

1. Problem personnel are dealt with constructively by our Disagree Strongly administration. Disagree Slightly

Neutral

Agree Slightly Agree Strongly Not Applicable

1. I get adequate, timely info about events that might Disagree Strongly affect my work, from the leadership. Disagree Slightly

Neutral

Agree Slightly Agree Strongly Not Applicable

1. The levels of staffing in this clinical area are Disagree Strongly

sufficient to handle the number of patients. Disagree Slightly Neutral

Agree Slightly Agree Strongly Not Applicable

1. This transplant center does a good job of training new Disagree Strongly personnel. Disagree Slightly

Neutral

Agree Slightly Agree Strongly Not Applicable

1. All the necessary information for diagnostic and Disagree Strongly

therapeutic decisions is routinely available to me. Disagree Slightly Neutral

Agree Slightly Agree Strongly Not Applicable

1. Trainees in my discipline are adequately supervised. Disagree Strongly

Disagree Slightly Neutral

Agree Slightly Agree Strongly Not Applicable

1. I experience good collaboration with nurses in my Disagree Strongly clinical area. Disagree Slightly

Neutral

Agree Slightly Agree Strongly Not Applicable

1. I experience good collaboration with staff physicians Disagree Strongly in my clinical area. Disagree Slightly

Neutral

Agree Slightly Agree Strongly Not Applicable

1. I experience good collaboration with pharmacists in my Disagree Strongly clinical area. Disagree Slightly

Neutral

Agree Slightly Agree Strongly Not Applicable

1. I experience good collaboration with social workers in Disagree Strongly my clinical area Disagree Slightly

Neutral

Agree Slightly Agree Strongly Not Applicable

1. Communication breakdowns that lead to delays in Disagree Strongly delivery of care are common in my clinical area. Disagree Slightly

Neutral

Agree Slightly Agree Strongly Not Applicable

1. Position: (Mark only one that matches closest) Attending/Staff Physician Fellow Physician Resident Physician

Nurse Manager/Charge Nurse Registered Nurse

Pharmacist

Therapist (RT, PT, OT, Speech) Clinical Social Worker Dietician/Nutritionist

Clinical Support (CMA, EMT, Nurses Aide, etc) Technologist/Technician (e.g. Surg, Lab, Rad) Admin Support (Clerk, Secretary, Receptionist) Environmental Support (Housekeeper)

Other Management (Administration, Clinic Manager) Advanced Transplant Provider (e.g NP, PA)

Other (Not covered above)

1. Which organ group closely aligns to your job Heart responsibilities? Kidney

Liver Lung Multiple Other

1. Time in your current job: Less than 6 months 6-11 months

1-2.99 years

3-4.99 years

5-10.99 years

11-20.99 years 21 or more years

1. Mark your gender Male

Female

1. Have you filled out this survey before? Yes No
